# Comprehensive mendelian randomization reveals atrial fibrillation-breast cancer relationship and explores common druggable targets

**DOI:** 10.3389/fphar.2024.1435545

**Published:** 2024-08-07

**Authors:** Fenglin Qi, Lunzhe Yang, Guanglei Chang, Xiangbin Wang, Guanghong Tao, Hua Xiao

**Affiliations:** ^1^ Department of Cardiology, The First Affiliated Hospital of Chongqing Medical University, Chongqing, China; ^2^ Department of Neurosurgery, Guangdong Provincial People’s Hospital (Guangdong Academy of Medical Sciences), Southern Medical University, Guangzhou, China; ^3^ School of Life Sciences and Biopharmaceutics, Guangdong Pharmaceutical University, Guangzhou, China

**Keywords:** atrial fibrillation, breast cancer, mendelian randomization, drug target, cardio-oncology

## Abstract

**Background:**

Atrial fibrillation (AF) and breast cancer pose significant risks to human health. The reasons behind the concurrent occurrence of AF and breast cancer remain unclear, leading to complex treatment approaches. Mendelian Randomization (MR) analyses aim to offer genetic evidence supporting the causation of AF and breast cancer and to investigate common druggable genes associated with both conditions.

**Methods:**

We used two-samples of MR to sequentially explore the causal relationship between atrial fibrillation and breast cancer, and between atrial fibrillation and breast cancer therapeutic drugs, and verified the stability of the results through colocalization analysis. We utilized the Connectivity map database to infer the direction of drug effects on disease. Finally, we explored druggable genes that play a role in AF and breast cancer and performed a Phenome-wide MR analysis to analyze the potential side effects of drug targets.

**Results:**

We found 15 breast cancer therapeutic drugs that significantly support a causal association between AF and breast cancer through expression in blood and/or atrial appendage tissue. Among these, activation of *ANXA5* by Docetaxel, inhibition of *EIF5A* by Fulvestrant, and inhibition of *GNA12* by Tamoxifen increased the risk of AF, while inhibition of *ANXA5* by Gemcitabine and Vinorebine and inhibition of *PCGF6* by Paclitaxel reduced the risk of AF. Inhibition of *MSH6* and *SF3B1* by Cyclophosphamide, as well as inhibition of *SMAD4* and *PSMD2* and activation of *ASAH1* and *MLST8* by Doxorubicin can have bidirectional effects on AF occurrence. *XBP1* can be used as a common druggable gene for AF and breast cancer, and there are no potential side effects of treatment against this target.

**Conclusion:**

This study did not find a direct disease causality between AF and breast cancer but identified 40 target genes for 15 breast cancer therapeutic drugs associated with AF, clarified the direction of action of 8 breast cancer therapeutic drugs on AF, and finally identified one common druggable target for AF and breast cancer.

## Introduction

Atrial fibrillation (AF) is the most common cardiac arrhythmia associated with heart failure, stroke, and death (Zoni-Berisso et al., 2014). Studies have shown that the majority of deaths in patients with AF are not due to arrhythmia but to concomitant complications or comorbidities, including malignancy, and most oncological therapies are also considered to be high-risk factors for arrhythmia (Harbeck and Gnant, 2017; Bisbal et al., 2020). Breast cancer is the most common malignancy in women and one of the three most common cancers worldwide (Madnick and Fradley, 2022). Cardiovascular disease is increasingly becoming a significant prognostic barrier for breast cancer patients (Galimzhanov et al., 2023). Despite two mentions to cancer in the 2020 ESC guidelines on treating and managing AF, little guidance is provided for this area (Hindricks et al., 2021). Disease coexistence in AF combined with breast cancer is common, but the reasons for their coexistence are less well explored and treatment is in limbo.

Currently, studies on the association between atrial fibrillation and breast cancer are mainly based on epidemiologic surveys or retrospective data statistics. Several studies and meta-analyses have shown that the incidence of atrial fibrillation is increased in patients with breast cancer, and female patients with atrial fibrillation have an increased risk of breast cancer. Still, the correlation between the two diseases has not yet been established (Hung et al., 2019; Yun et al., 2021; Guha et al., 2022; Yao et al., 2023). The increased incidence of AF may be potentially associated with medications related to cancer treatment in addition to the cancer itself (Merino, 2022). Thus, there is no consistent conclusion as to whether the increased incidence of AF is more related to cancer itself or oncologic therapies. Since the evidence for these studies is based on observational studies, it is not possible to prove a causal relationship between them.

Advances in genetics have significantly shaped the pursuit of causality. Mendelian randomization (MR), a genetic analysis, employs Single-nucleotide polymorphisms (SNPs) from genome-wide association studies (GWAS) as instrumental variables (IVs) to establish causal links between exposures and outcomes. Genetic variation due to random assignment of alleles makes the results less susceptible to reverse causation and confounding bias (Davey Smith and Hemani, 2014). Proteins are pivotal in diverse biological processes and are key targets for drug development (Zheng et al., 2020). Nelson et al. (2015) demonstrated that a protein drug target is twice as likely to receive market approval if its link to disease is supported by genetic association. In recent years, MR analysis has been extensively applied in the development and repurposing of drug targets. Within drug target MR studies, SNPs with significant impacts on protein products within a specific genomic vicinity of the target gene, such as expression quantitative trait loci (eQTLs) and protein quantitative trait loci (pQTLs), serve as IVs in combination with GWAS datasets related to the disease under investigation to scrutinize the target’s genetic influence on the disease (Schmidt et al., 2020). Currently, there are no extensive studies analyzing the relationship between AF and breast cancer by systematic MR analysis, nor are there drug-targeted MR analyses analyzing the association between these two diseases and drugs, as well as exploring potentially druggable genes that play a common role in these two diseases.

In this study, we conducted systematic Mendelian Randomization (MR) analysis utilizing eQTLs identified in blood and atrial appendage tissues, coupled with genetic datasets related to atrial fibrillation (AF) and breast cancer. We aimed to explore the causal relationships and potential associations with drugs between these two diseases and identify novel shared therapeutic targets.

## Materials and methods

### Study design

**FIGURE 1:**
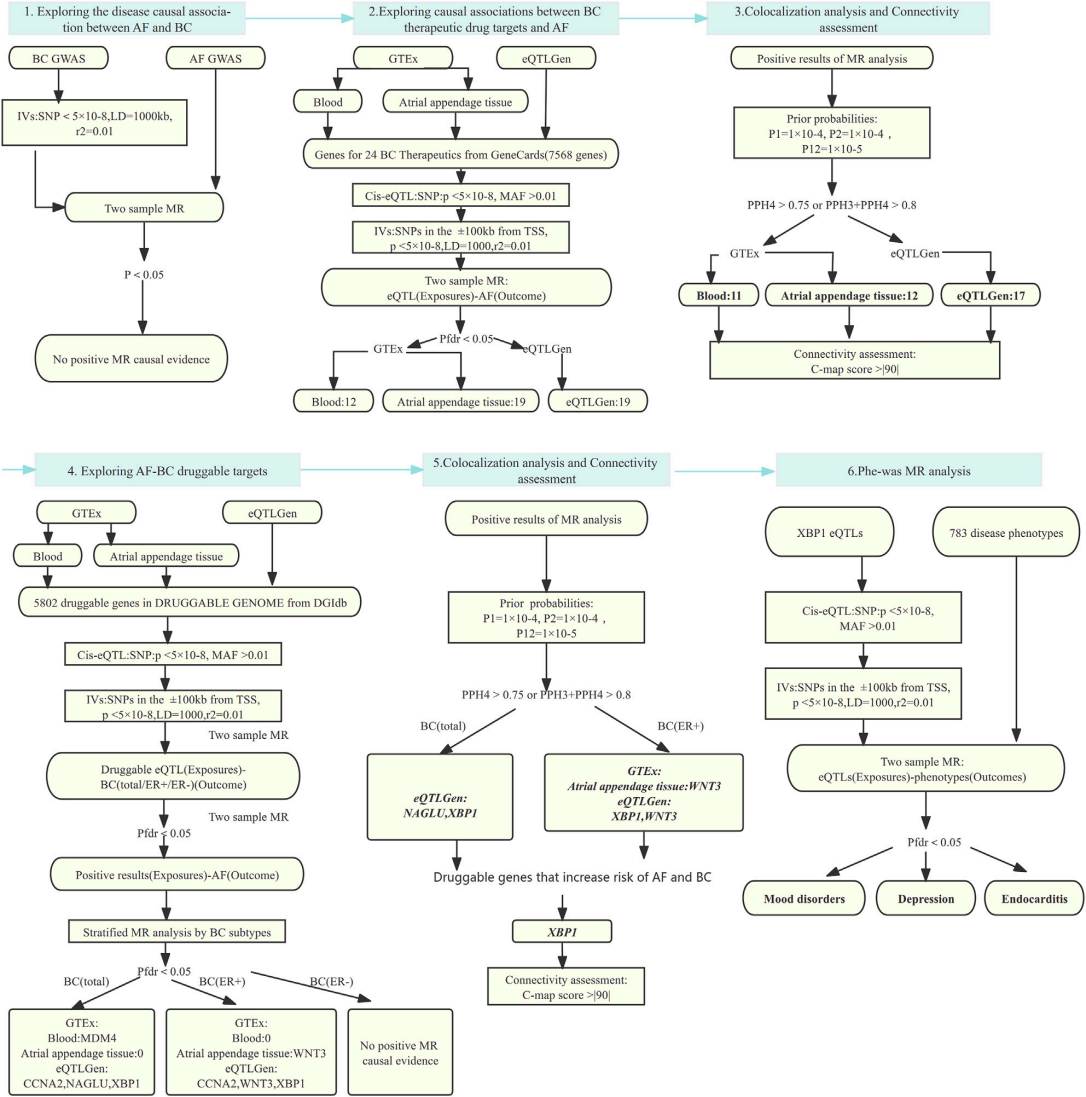
MR analysis and colocalization analysis process of the study. MR: Mendelian randomization, AF: Atrial fibrillation, BC: Breast cancer, BC(total/ER+/ER-):Breast cancer (total cases, estrogen receptor positive, estrogen receptor negative), eQTL: Expression quantitative trait locus, IVs: Instrumental variables, SNP: Single nucleotide polymorphism, MAF: Minor allele frequency; TSS: transcriptional start site; LD: Linkage disequilibrium; Pfdr: False Discovery Rate corrected P-value; PP: Posterior probability, GTEx: Genotype-Tissue Expression project, eQTLGen: eQTLGen Consortium, C-map: Connectivity map.

We performed the analysis by the following steps: 1) Analyze the effect of breast cancer on the risk of AF using two-sample MR analysis, and further perform colocalization analysis if a causal effect exists to verify the robustness of the results. 2) The effect of 24 breast cancer therapeutic drug targets on AF was analyzed using two-sample MR analysis, and the results obtained were also subjected to colocalization analysis. Then, we utilized the Connectivity-map (C-map) dataset to infer the direction of drug effects on disease risk from the obtained robust results. 3) Based on the druggable gene data, two-sample MR and colocalization analyses were used to explore the positive targets that play a role in AF and breast cancer. Finally, Phenome-wide MR analysis was performed to explore whether the resulting genes as drug targets were accompanied by potential side effects. The process of this study is shown in Figure 1.

The three core assumptions of MR were followed: 1) IVs are strongly associated with exposure, 2) IVs are not associated with potential confounders affecting the exposure-outcome interval, and 3) IVs affect outcomes only through exposure.

### AF and breast cancer datasets

AF data were obtained from a large-scale GWAS meta-analysis based on the Atrial Fibrillation Genetics (AFGen) consortium results and the majority of the Broad AF Study (Broad AF), which included over half a million individuals. We selected a population of European ancestry from this database for our study data, including 55,114 AF cases and 482295 control individuals (Roselli et al., 2018). Breast cancer data were obtained from the GWAS of 228,951 women of European ancestry provided by the Breast Cancer Association Coalition (BCAC), including 122977 breast cancer cases (69,501 ER+ and 21,468 ER-) and 105974 control individuals (Michailidou et al., 2017). Breast cancer data were stratified and analyzed for different subcategories (total, ER+, ER-) (Supplementary Table S1).

### Breast cancer treatment drug genetics and druggable genes

GeneCards (V.5.19) is a human genes compendium that integrates 466,053 gene data from 150 sources to provide genomic, proteomic, transcriptomic, genetic, and functional information on all known and predicted human genes (Stelzer et al., 2016). We selected 24 breast cancer therapeutic drugs based on the guidelines, involving chemotherapy, endocrine therapy, immunotherapy, and targeted therapy (Korde et al., 2021). 24 common breast cancer therapeutic drugs were entered sequentially into the GeneGards website to find the targets of these drugs, and eventually, the drug targets of these drugs were summarized (Supplementary Table S2).

The Drug-Gene Interaction Database (DGIdb V.5.0.4) summarizes drug-gene interactions from existing studies and multiple drug-gene databases, and it includes 43 potentially druggable gene categories and at least 30 interaction types as defined by source datasets. A target is considered druggable if it is categorized in the DRUGGABLE GENOME in the drug information provided by DGIdb, and a total of 5,802 druggable genes were identified in the DGIdb database (Cannon et al., 2024).

### Genetic datasets

Cis-eQTL are located within the region of the regulated gene itself, such as the promoter and coding region of a gene, proximal to the regulated gene (usually limited to within 1 Mb on either side of the coding gene). The eQTL dataset used in this study was obtained from the Genotype-Tissue Expression project (GTEx) Version eight and the eQTLGen consortium. eQTL datasets were obtained from the GTEx database by determining 15,201 RNA sequencing samples extracted from 49 tissues from 838 *postmortem* donors (85% from European populations) (Consortium, 2020). The eQTLGen data result from a large-scale meta-analysis of up to 31,684 blood samples from 37 eQTLGen consortium cohorts (Võsa et al., 2021). Most drugs act through the blood, so we chose blood samples from two consortia cis-eQTL as the IVs for our study. In addition, the atrial appendage tissue was found to be the source of ectopic triggering and reentrant atrial tachycardia in some patients with atrial fibrillation. Its remodeling provides for the formation and maintenance of AF drivers (Schuessler et al., 1993; Di Biase et al., 2010). Therefore, we also selected cis-eQTL data of atrial appendage tissue from GTEx to explore the tissue specificity of drug action. Detailed links to the database have been placed in Supplementary Table S1.

### Principle of colocalization analysis

Colocalization analysis is used to test whether two phenotypes share the same causal variant within a given region, which can provide indicative evidence of overlapping genetic mechanisms between two phenotypes. There are five hypotheses for colocalization analysis:H0: all SNPs within an area are not significantly associated with phenotype 1 and phenotype 2, H1/H2: there are SNPs within a region that are significantly associated with either phenotype 1 or phenotype 2, H3: there are SNPs within a region that are significantly associated with both phenotype 1 and phenotype 2, but they are different, and H4: there are SNPs within a region that are significantly associated with both phenotype 1 and phenotype 2, and they are the same SNPs (Giambartolomei et al., 2014).

### The causal relationship between atrial fibrillation and breast cancer

We used R software and Two Sample MR code package V.0.5.8 for analysis. First, breast cancer (total, ER+, ER-) GWAS was used as an exposure, from which eligible SNPs were selected as IVs based on the criterion that the *p*-value of the SNPs was <5 × 10^−8^. First, breast cancer (total, ER+, ER-) GWAS were used as an exposure, from which eligible IVs were selected based on the criterion that the *p*-value of the SNP < 5 × 10^−8^. To avoid linkage disequilibrium (LD) and to ensure independence, the window of LD was set to 10,00 kb, r2 = 0.01 (based on 1,000 Genomes European Reference Panel) (Abecasis et al., 2012; Michailidou et al., 2017). We removed SNPs that could not be matched or had palindromic sequences. MR analyzed the filtered IVs with AF GWAS data. The inverse variance weighting (IVW) method was selected as the primary analysis method when there were multiple SNPs, and the Wald ratio method was chosen as the analysis method if there was only one SNP. Meanwhile, we performed sensitivity analyses to test the reliability of the results, using weighted median, weighted mode, MR-Egger regression, and MR-PRESSO methods under different conditions (Burgess et al., 2019). The presence of horizontal pleiotropy was determined based on the MR-Egger regression intercept and MR-PRESSO outlier correction (Bowden et al., 2015; Verbanck et al., 2018). The presence of heterogeneity was assessed using Cochran’s Q statistic, and the presence of heterogeneity was indicated when the *p*-value was <0.05. In addition, the leave-one-out analysis was performed by removing each SNP to assess the robustness of the MR results further. The results were optimistic when the obtained *p*-value was <0.05.

The obtained positive results were subjected to colocalization analysis to verify the stability of the results. Based on the above principles of colocation analysis, we selected a gene range of ± 1,000 kb as the region for colocalization analysis. We performed the colocalization analysis on the obtained MR analysis results using the R package COLOC V.5.2.3 and the prior probabilities P1 = 1 × 10^−4^, P2 = 1 × 10^−4^, and P12 = 1 × 10^−5^. P1, P2 and P12 denote the likelihood that the SNPs are significantly associated with the eQTL, the disease (breast cancer or atrial fibrillation) or both, respectively. The posterior probability (PP) and the likelihood of the five hypotheses were calculated using the Bayesian test, and finally, the results were visualized by Locus CompareR (Giambartolomei et al., 2014; Liu B. et al., 2019). When PPH4 > 0.75 or PPH3+PPH4 > 0.8, it was considered as the target positive result of our study.

### Effect of clinical therapeutic drugs for breast cancer on atrial fibrillation

We used GeneCards to screen target genes for 24 breast cancer therapeutic drugs. Then we selected cis-eQTL of these genes as IVs from GTEx and eQTLGen, respectively, with the criteria of *p*-value <5 × 10^−8^, distance from the transcriptional start site (TSS) of each gene ± 100 kb, and MAF (minor allele frequency) > 0.01 (Zhu et al., 2016; Consortium, 2020; Võsa et al., 2021). GWAS of atrial fibrillation was used as an outcome, integrating and analyzing eQTL data with GWAS data to determine causality (Abecasis et al., 2012; Bowden et al., 2015; Burgess et al., 2019). We corrected the results for multiple comparisons using a false discovery rate (FDR) correction, which reduces the false-positive rate, and a *p*-value (Pfdr) < 0.05 consistent with the FDR correction was considered a positive result. Finally, we performed colocalization analysis and indicated the results to be robust when the resulting PPH4 > 0.75 or PPH3+PPH4 > 0.8.

### Direction determination of drug-drug target-disease effect

C-map is a linkage tool for discovering small molecules, chemical and physiological processes that share mechanisms of action as well as relationships between disease, genetic perturbation, and drug action (Lamb et al., 2006). From the C-map database, we could obtain 1) changes in the expression levels of genes after drug use and 2) connectivity scores for gene overexpression and gene knockdown.

The connectivity score in the C-map database can be positive (similar expression pattern) or negative (opposite expression pattern). We set a C-map score > |90| as our outcome criterion. Suppose the high expression of a gene is found to increase the risk of atrial fibrillation, and a breast cancer therapeutic drug is given with a pharmacologic effect similar to the promotion of overexpression of that gene. In that case, it is inferred that the drug increases the risk of atrial fibrillation in the treatment of breast cancer. Conversely, if it is similar to knocking down the expression of that risk gene, it is inferred that the drug is expected to reduce the risk of AF and become a possible AF therapeutic drug.

### Exploring common drug targets in AF-breast cancer

Based on the 5,802 druggable genes summarized in DGIdb, cis-eQTL that met the criteria in GTEx and eQTLGen were selected as IVs(*p*-value <5 × 10^−8^, distance from the transcriptional start site (TSS) of each gene ± 100kb, and MAF (minor allele frequency) > 0.01), and breast cancer GWAS (total, ER+, ER-) were used as the outcomes for two-sample MR analysis. Similarly, a two-sample MR analysis was performed with the previously obtained positive targets as exposures and AF GWAS as result. FDR correction and colocalization analyses were performed on the results obtained, and results meeting Pfdr <0.05 and PPH4 >0.75 or PPH3 + PPH4 >0.8 were considered positive targets. The parameter settings for two sample MR analysis and colocalization analysis were as before.

### Phenome-wide MR analysis

Considering the possibility of drug safety, we performed a Phenome-Wide MR Analysis with the resulting AF-breast cancer drug targets as exposures and the disease phenotypes summary data as the outcomes to investigate whether the resulting targets used to reduce the risk of AF and breast cancer were accompanied by potential side effects. Disease phenotype data from the United Kingdom biobank contained 408,961 participants with >1,400 binary phenotypes. Due to statistical efficacy, we filtered phenotypes with cases <500 and ultimately included 783 disease phenotype data (Zhou et al., 2018). The selection of IVs, setting parameters, and criteria for validation of results were consistent with the preliminary analysis.

## Results

### Impact of breast cancer risk on AF incidence

We did not find MR evidence of a causal association between genetic predisposition to breast cancer (total, ER+, ER-) and the occurrence of AF (OR: 0.99,95% CI, 0.97–1.02, *P* = 0.58, OR:1.01,95% CI,0.98–1.04, *P* = 0.42, OR:0.99,95%CI, 0.94–1.04, *P* = 0.62). (Supplementary Tables S3–S5).

### Effect of breast cancer therapeutic drugs on the risk of atrial fibrillation

**FIGURE 2:**
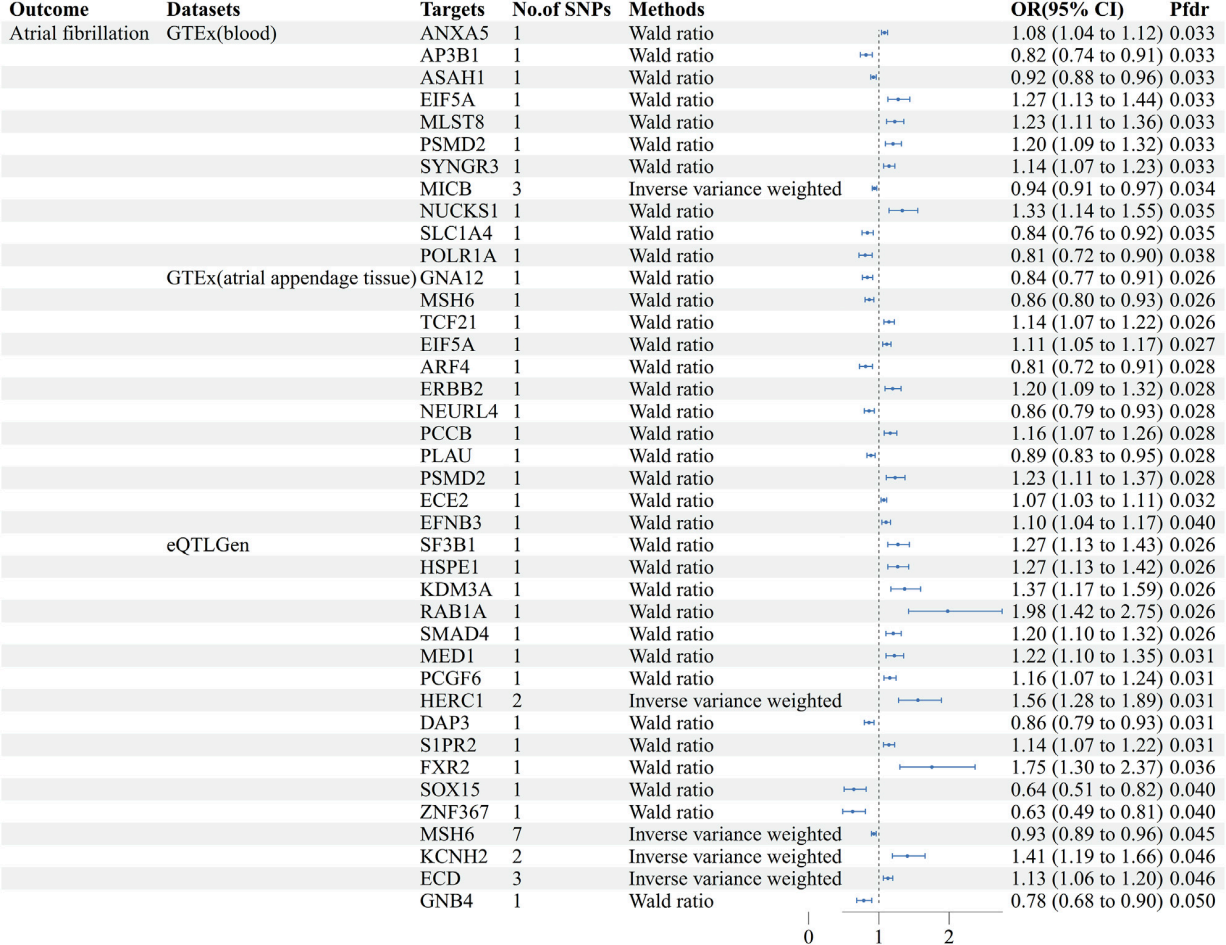
Forest plots of causal associations between AF and breast cancer treatment drugs. MR: Mendelian randomization, No. Of SNPs: Number of SNPs, OR: Odd ratio, CI: Confidence interval, Pfdr: False discovery rate corrected P-value, GTEx: Genotype-Tissue Expression project, eQTLGen: eQTLGen Consortium.

Using GeneCards, we screened 7,568 drug target genes for 24 breast cancer therapeutic drugs. After two-sample MR analysis and colocalization analysis, we obtained 40 positive results (PPH4 > 0.75 or PPH3+PPH4 > 0.8): GTEx (blood): 11, GTEx (atrial appendage tissue):12,eQTLGen: 17 (Table 1; Figure 2). According to statistical principles, in the results of Pfdr < 0.05 obtained from the MR analysis of the drug target and AF, OR > 1 was categorized as having a pro-AF occurrence effect, and OR < 1 as having an AF-protective effect. Of these, 23 targets were statistically correlated with the occurrence of atrial fibrillation, and 14 targets had AF-protective effects. Categorizing the targets showed that 15 drugs affected AF by each passing through several targets (Table 2). Our MR analysis did not identify an additional nine drugs that affected AF (Supplementary Tables S6–S10).

**TABLE 1:** Colocalization analysis of positive MR results.

Outcome	eQTL datasets	Gene	No.of SNPs	PPH0	PPH1	PPH2	PPH3	PPH4
AF	GTEx (blood)	ANXA5	746	6.91E-48	1.65E-01	4.82E-49	1.07E-02	8.24E-01
		AP3B1	791	6.95E-13	1.77E-01	6.48E-14	1.57E-02	8.07E-01
		ASAH1	2,015	1.48E-93	9.03E-12	1.64E-82	1.00E+00	3.85E-11
		EIF5A	373	2.92E-08	5.14E-02	5.88E-08	1.03E-01	8.46E-01
		MLST8	450	6.09E-13	6.11E-04	9.92E-10	9.95E-01	3.95E-03
		PSMD2	661	5.38E-16	1.22E-01	1.87E-15	4.24E-01	4.54E-01
		SYNGR3	471	6.48E-16	1.11E-07	5.86E-09	1.00E+00	1.02E-06
		MICB	5,195	4.52E-35	5.55E-02	3.05E-34	3.74E-01	5.71E-01
		NUCKS1	495	6.93E-08	3.16E-03	1.67E-05	7.61E-01	2.36E-01
		SLC1A4	459	2.18E-08	1.95E-01	1.63E-09	1.39E-02	7.91E-01
		POLR1A	1,050	8.76E-08	1.07E-01	4.27E-07	5.23E-01	3.70E-01
	GTEx (atrial appendage tissue)	GNA12	698	4.73E-05	3.81E-03	1.15E-03	9.18E-02	9.03E-01
		MSH6	777	7.99E-06	1.23E-01	1.23E-06	1.81E-02	8.59E-01
		TCF21	569	1.69E-05	7.38E-02	1.82E-05	7.85E-02	8.48E-01
		EIF5A	740	5.22E-18	9.06E-02	1.39E-17	2.40E-01	6.69E-01
		ARF4	1,018	2.85E-05	1.98E-01	3.15E-05	2.19E-01	5.82E-01
		ERBB2	669	2.54E-05	3.01E-02	2.36E-04	2.80E-01	6.89E-01
		NEURL4	422	1.26E-05	1.94E-01	2.64E-05	4.04E-01	4.02E-01
		PCCB	1,173	3.12E-09	7.81E-02	3.06E-09	7.56E-02	8.46E-01
		PLAU	896	6.37E-23	3.69E-20	1.72E-03	9.97E-01	8.75E-04
		PSMD2	447	1.29E-05	1.18E-01	4.49E-05	4.11E-01	4.71E-01
		ECE2	692	9.50E-11	1.57E-01	3.42E-10	5.65E-01	2.78E-01
		EFNB3	361	5.19E-06	8.37E-04	6.15E-03	9.91E-01	2.40E-03
	eQTLGen	ENSG00000115524	1,855	9.86E-50	4.49E-02	1.29E-48	5.87E-01	3.68E-01
		ENSG00000115541	1,095	7.75E-17	4.63E-02	8.40E-16	5.01E-01	4.52E-01
		ENSG00000115548	1,205	1.76E-30	4.42E-05	3.98E-26	1.00E+00	4.02E-04
		ENSG00000138069	464	2.68E-17	3.02E-14	3.85E-04	4.34E-01	5.65E-01
		ENSG00000141646	1,285	2.05E-87	3.50E-02	8.08E-87	1.37E-01	8.28E-01
		ENSG00000125686	1,624	1.46E-68	6.57E-02	1.45E-67	6.52E-01	2.82E-01
		ENSG00000156374	1,630	5.30E-85	5.07E-41	1.05E-44	1.00E+00	1.91E-40
		ENSG00000103657	2,149	1.26E-59	1.63E-03	6.69E-57	8.60E-01	1.38E-01
		ENSG00000132676	1,588	1.09E-163	4.01E-51	2.70E-113	1.00E+00	1.80E-50
		ENSG00000175898	579	2.80E-44	1.93E-01	1.25E-45	7.82E-03	7.99E-01
		ENSG00000129245	471	5.00E-09	4.43E-06	1.13E-03	9.99E-01	1.34E-04
		ENSG00000129194	752	3.60E-13	3.16E-06	1.14E-07	1.00E+00	1.68E-05
		ENSG00000165244	765	2.28E-10	1.32E-02	3.17E-09	1.83E-01	8.04E-01
		ENSG00000116062	2,689	6.69E-241	1.50E-01	4.11E-241	9.15E-02	7.58E-01
		ENSG00000055118	1,302	2.99E-71	3.28E-06	4.86E-67	5.24E-02	9.48E-01
		ENSG00000122882	2,449	0.00E+00	5.32E-21	3.75E-300	1.00E+00	1.30E-18
		ENSG00000114450	917	1.14E-38	9.50E-04	1.20E-35	9.98E-01	1.22E-03

MR:mendelian randomization, AF:Atrial fibrillation, GTEx:Genotype-Tissue Expression project, eQTL:expression quantitative trait locus,No.of SNPs:Number of SNPs,PP:Posterior probability.

**TABLE 2:** Positive results of breast cancer treatment drugs on atrial fibrillation.

Drugs	Outcome	Targets			Total	Criteria
		GTEX (blood)	GTEX (atrial appendage tissue)	eQTLGen		
Cisplatin	Atrial fibrillation	ANXA5,ASAH1,NUCKS1,POLR1A	TCF21,EIF5A,ARF4,NEURL4,PCCB	SF3B1,HSPE1,KDM3A,RAB1A,MED1, S1PR2,SOX15,KCNH2,ECD	18	Pfdr < 0.05, Q-pval > 0.05, Pleiotropy-pval > 0.05, PPH4 > 0.75 or PPH3 + PPH4 > 0.8
Tamoxifen		ANXA5,EIF5A	GNA12,EIF5A,ECE2	KDM3A,SMAD4,MED1,PCGF6,HERC1,FXR2,ZNF367,KCNH2,ECD,GNB4	15	
Doxorubicin		ANXA5,ASAH1,MLST8,PSMD2,SLC1A4	GNA12,PSMD2,EFNB3	SF3B1,HSPE1,SMAD4,DAP3,KCNH2	13	
Paclitaxel		ANXA5,AP3B1,ASAH1,SYNGR3,NUCKS1,SLC1A4		SMAD4,PCGF6,SOX15,KCNH2	10	
Gemcitabine		ANXA5,ASAH1,EIF5A,MICB	MSH6,EIF5A,ERBB2,PLAU	SMAD4,MSH6	10	
Fulvestrant		EIF5A,MICB, POLR1A	EIF5A,ERBB2,PLAU	MED1,FXR2,GNB4	9	
Docetaxel		ANXA5,ASAH1	MSH6,ERBB2,PLAU	MSH6	6	
Capecitabine			MSH6,ERBB2,PLAU	SMAD4,MED1,MSH6	6	
Cyclophosphamide		ANXA5	MSH6,ERBB2,PLAU	SF3B1,MSH6	6	
Trastuzumab		ANXA5		SF3B1,KCNH2	3	
Vinorelbine, Epirubicin		ANXA5			1	
Neratinib, Palbociclib,Letrozole			ERBB2		1	

MR:mendelian randomization, GTEx:Genotype-Tissue Expression project, Pfdr:False discovery rate corrected *P*-value, Q-pval:The *p*-value of Cochran’s Q, Pleiotropy-pval:The *p*-value for horizontal pleiotropy, PP:Posterior probability.

### Exploring the direction of breast cancer therapeutics acting on AF

**FIGURE 3:**
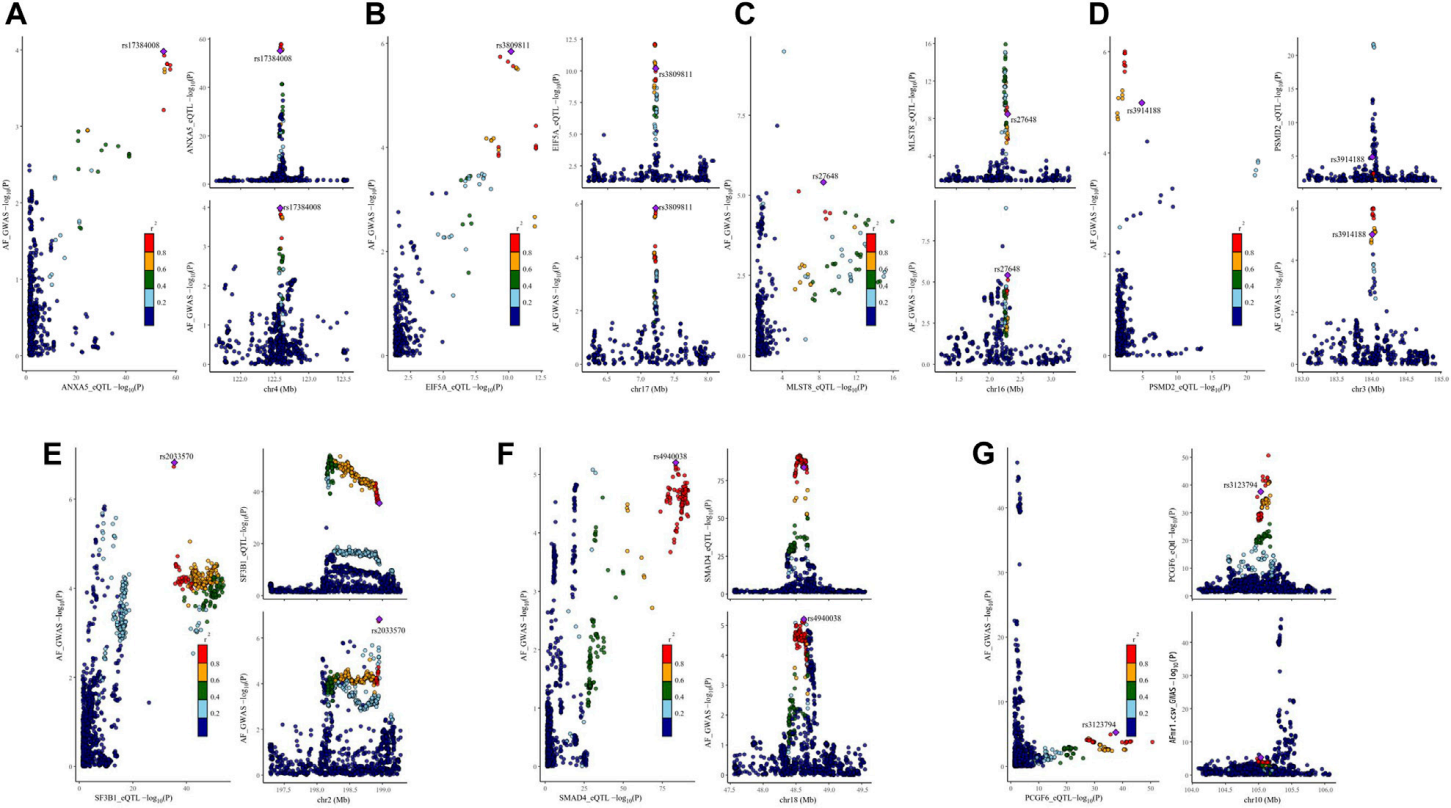
Visualization of the results of colocalization analysis of breast cancer therapeutic drug targets and AF occurrence. The plot on the left represents the distribution of SNPs in eQTL and GWAS at -log10(p), and the two plots on the right represent the distribution of SNPs in eQTL and GWAS, respectively. AF: Atrial fibrillation, r2: degree of association between SNPs and top SNPs. **(A)** colocalization analysis of ANXA5 and AF **(B)** colocalization analysis of EIF5A and AF **(C)** colocalization analysis of MLST8 and AF **(D)** colocalization analysis of PSMD2 and AF **(E)** colocalization analysis of SF3B1 and AF **(F)** colocalization analysis of SMAD4 and AF **(G)** colocalization analysis of PCGF6 and AF.

Based on the C-map database, we obtained the direction of action and connectivity map score (C-map score >|90|) of eight breast cancer therapeutic drugs on 10 target genes and then explored the effects of these drugs on AF. (Table 3) seven targets that are risk targets for causing the development of AF, and we visualized their colocalization results with AF (Figure 3). Activation of ANXA5 by Docetaxel (score:91.44), inhibition of EIF5A by Fulvestrant (score: 94.15), and inhibition of GNA12 by Tamoxifen (score:93.41) increased the risk of AF. Inhibition of ANXA5 by Gemcitabine (score:98.78) and Vinorebine (score:94.29) and inhibition of PCGF6 by Paclitaxel (score:98.70) reduced the risk of AF. Among them, Tamoxifen can act through the target of atrial appendage tissue, and the rest of the drugs act through blood circulation. In addition, since multiple targets in different tissues can be acted upon, we found that Cyclophosphamide increased the risk of AF when it inhibited MSH6(score:93.04) via blood and atrial appendage tissue. At the same time, it reduced the risk of AF when it inhibited SF3B1(score:90.13) via blood. The use of Doxorubicin also had effects on AF in different directions, reducing the risk of AF by circulatory inhibition of SMAD4 (score:95.60) and activation of ASAH1 (score:94.57) and reducing the risk of AF by inhibition of PSMD2(score:94.04) via the bloodstream and atrial appendage tissue, and by activation of MLST8 (score:90.99) via the bloodstream increasing the risk of AF (Supplementary Table S10).

**TABLE 3:** Directional assessment of the impact of target genes of breast cancer therapeutics on atrial fibrillation.

Drugs	Targets	Outcome	eQTL datasets	Method	No.of SNPs	OR (95%CI)	Pfdr	PPH3	PPH4	PPH3 + PPH4	Perturbagen Type	Connectivity score
Docetaxel	ANXA5	Atrial fibrillation	GTEx (blood)	Wald ratio	1	1.08 (1.04–1.12)	3.35E-02	1.07E-02	8.24E-01	8.35E-01	Gene Over-Expression	91.44
Fulvestrant	EIF5A		GTEx (blood)	Wald ratio	1	1.27 (1.13–1.44)	3.35E-02	2.40E-01	6.69E-01	9.09E-01	Gene Knock-Down	−94.15
Tamoxifen	GNA12		GTEx (atrial appendage tissue)	Wald ratio	1	0.84 (0.77–0.91)	2.64E-02	8.90E-01	7.38E-02	9.64E-01	Gene Knock-Down	93.41
Gemcitabine	ANXA5		GTEx (blood)	Wald ratio	1	1.08 (1.04–1.12)	3.35E-02	1.07E-02	8.24E-01	8.35E-01	Gene Knock-Down	98.78
Vinorelbine	ANXA5		GTEx (blood)	Wald ratio	1	1.08 (1.04–1.12)	3.35E-02	1.07E-02	8.24E-01	8.35E-01	Gene Knock-Down	94.29
Paclitaxel	PCGF6		eQTLGen	Wald ratio	1	1.16 (1.07–1.24)	3.13E-02	1.00E+00	1.91E-40	1.00E+00	Gene Knock-Down	98.70
Cyclophosphamide	MSH6		GTEx (atrial appendage tissue)	Wald ratio	1	0.86 (0.80–0.93)	2.64E-02	1.81E-02	8.59E-01	8.77E-01	Gene Knock-Down	93.04
	MSH6		eQTLGen	Inverse-variance weighted	7	0.93 (0.89–0.96)	4.48E-02	9.15E-02	7.58E-01	8.50E-01	Gene Knock-Down	93.04
	SF3B1		eQTLGen	Wald ratio	1	1.27 (1.13–1.43)	2.62E-02	5.87E-01	3.68E-01	9.55E-01	Gene Knock-Down	90.13
Doxorubicin	SMAD4		eQTLGen	Wald ratio	1	1.20 (1.10–1.32)	2.62E-02	1.37E-01	8.28E-01	9.65E-01	Gene Knock-Down	95.60
	ASAH1		GTEx (blood)	Wald ratio	1	0.92 (0.88–0.96)	3.35E-02	1.00E+00	3.85E-11	1.00E+00	Gene Over-Expression	94.57
	PSMD2		GTEx (blood)	Wald ratio	1	1.20 (1.09–1.32)	3.35E-02	4.11E-01	4.71E-01	8.82E-01	Gene Knock-Down	94.04
	MLST8		GTEx (blood)	Wald ratio	1	1.23 (1.11–1.36)	3.35E-02	9.95E-01	3.95E-03	9.99E-01	Gene Over-Expression	90.99

eQTL:expression quantitative trait locus, GTEx:Genotype-Tissue Expression project,No.of SNPs:Number of SNPs,OR:Odd ratio,CI:Confidence interval, Pfdr:False discovery rate corrected *P*-value,PP: posterior probability.

### Atrial fibrillation and breast cancer common druggable targets

**FIGURE 4:**
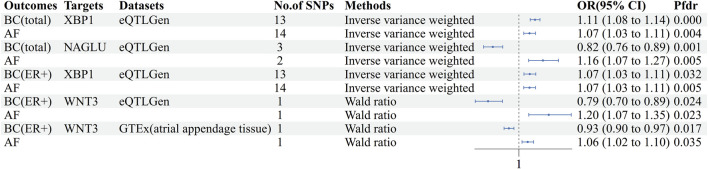
Forest plot of AF-BC druggable target exploration results. AF: Atrial fibrillation, BC: Breast cancer, BC(ER+):Breast cancer estrogen receptor positive, No. Of SNPs: Number of SNPs, OR: Odd ratio, CI: Confidence interval, Pfdr: False discovery rate corrected P-value, GTEx: Genotype-Tissue Expression project, eQTLGen: eQTLGen Consortium.

The cis-eQTL of 5,802 druggable genes were screened from GTEx and eQTLGen, respectively. Two-sample MR analysis was performed with druggable genes and breast cancer GWAS (total, ER+, ER-), and the results were analyzed with AF GWAS to obtain MR co-positive results. The co-positive results were then analyzed by colocalization with breast cancer GWAS and AF GWAS, respectively. The targets that colocalized positively with both diseases were selected as the final results (Supplementary Tables S11–S15). Two positive targets, XBP1 and NAGLU, were obtained based on the eQTLGen in the analysis of AF and breast cancer (total) (Figure 4). Among them, XBP1 had a genetically promoted incidence for both AF (OR = 1.07, 95% CI, 1.03–1.11, Pfdr = 0.004, PPH3+PPH4 = 0.90) and breast cancer (OR = 1.11,95%CI, 1.08–1.14, Pfdr = 2.41 × 10^−8^, PPH3+PPH4 = 1.00), NAGLU had a pro-incidence effect on AF (OR = 1.16,95%CI, 1.07–1.27, Pfdr = 0.005, PPH3+PPH4 = 0.81) and a protective effect on breast cancer (OR = 0.82,95%CI, 0.76–0.89, Pfdr = 0.001, PPH3+PPH4 = 1.00). In performing the analysis of AF with breast cancer (ER+), WNT3 and XBP1 were obtained based on eQTLGen. (Figure 4) WNT3 was associated with a risk of promoting the development of atrial fibrillation (OR = 1.20,95%CI, 1.07–1.35, Pfdr = 0.023, PPH3+PPH4 = 0.92), and was associated with a risk of protecting against breast cancer (OR = 0.79,95%CI, 0.70–0.89, Pfdr = 0.024, PPH3+PPH4 = 0.99), and XBP1 similarly had a promotional effect on the occurrence of atrial fibrillation (OR = 1.07,95%CI, 1.03–1.11, Pfdr = 0.005, PPH3+PPH4 = 0.90) and ER + breast cancer (OR = 1.07,95%CI, 1.03–1.11, Pfdr = 0.032, PPH3+PPH4 = 1.00). In GTEx (atrial appendage tissue), we obtained the WNT3 target, which had a similar promotional effect on the development of AF (OR = 1.06, 95% CI, 1.02–1.10, Pfdr = 0.035, PPH3+PPH4 = 0.98) and breast cancer (OR = 0.93, 95% CI, 0.90–0.97, Pfdr = 0.017, PPH3+PPH4 = 1.00) were consistent with the previous findings (Figure 4). We did not find MR evidence for targets that play a role in AF and breast cancer (ER-) (Table 4).

**TABLE 4:** Results of MR analysis and colocalization analysis of atrial fibrillation-breast cancer druggable genes.

Outcomes	Target	eQTL datasets	MR analysis (Inverse-variance weighted/Wald ratio)						Colocalization analysis		
			Method	No.of SNP	OR (95%CI)	Pfdr	Q-pval	Pleiotropy-pval	PPH3	PPH4	PPH3+PPH4
BC(total)	XBP1	eQTLGen	Inverse-variance weighted	13	1.11 (1.08–1.14)	2.41E-08	3.84E-01	5.95E-01	1.00E+00	1.00E-23	1.00E+00
AF			Inverse-variance weighted	14	1.07 (1.03–1.11)	4.42E-03	1.09E-01	4.86E-01	1.22E-01	7.73E-01	8.96E-01
BC(total)	NAGLU	eQTLGen	Inverse-variance weighted	3	0.82 (0.76–0.89)	1.50E-03	4.54E-01	5.62E-01	8.54E-01	1.45E-01	1.00E+00
AF			Inverse-variance weighted	2	1.16 (1.07–1.27)	5.49E-03	2.82E-01	NA	3.15E-02	7.75E-01	8.06E-01
BC(ER+)	XBP1	eQTLGen	Inverse-variance weighted	13	1.07 (1.03–1.11)	3.17E-02	2.29E-01	8.07E-01	1.00E+00	2.02E-19	1.00E+00
AF			Inverse-variance weighted	14	1.07 (1.03–1.11)	4.68E-03	1.09E-01	4.86E-01	1.22E-01	7.73E-01	8.96E-01
BC(ER+)	WNT3	eQTLGen	Wald ratio	1	0.79 (0.70–0.89)	2.38E-02	NA	NA	9.35E-01	5.84E-02	9.93E-01
AF			Wald ratio	1	1.20 (1.07–1.35)	2.26E-02	NA	NA	8.95E-01	2.67E-02	9.22E-01
BC(ER+)	WNT3	GTEx (atrial appendage tissue)	Wald ratio	1	0.93 (0.90–0.97)	1.70E-02	NA	NA	9.88E-01	1.10E-02	9.99E-01
AF			Wald ratio	1	1.06 (1.02–1.10)	3.45E-02	NA	NA	9.59E-01	1.82E-02	9.77E-01

MR:Mendelian randomization,ER+: Estrogen receptor positive, eQTL:expression quantitative trait locus,No.of SNPs:Number of SNPs,OR:Odd ratio,CI:Confidence interval, Pfdr:False discovery rate corrected P-value, Qpval:The *p*-value of Cochran’s Q, Pleiotropy-pval:The *p*-value for horizontal pleiotropy,PP:Posterior probability,NA:not available.

XBP1 was analyzed using C-map, which suggested the presence of 202 drugs that inhibit XBP1 expression (score:|>90|) with a similar effect as knockdown of the gene, which could reduce the risk of AF and breast cancer (total, ER+) (Supplementary Table S16). However, drug-gene information for NAGLU and WNT3 has not been obtained from C-map.

### Phenome-wide MR analysis of AF- breast cancer druggable gene

**FIGURE 5:**
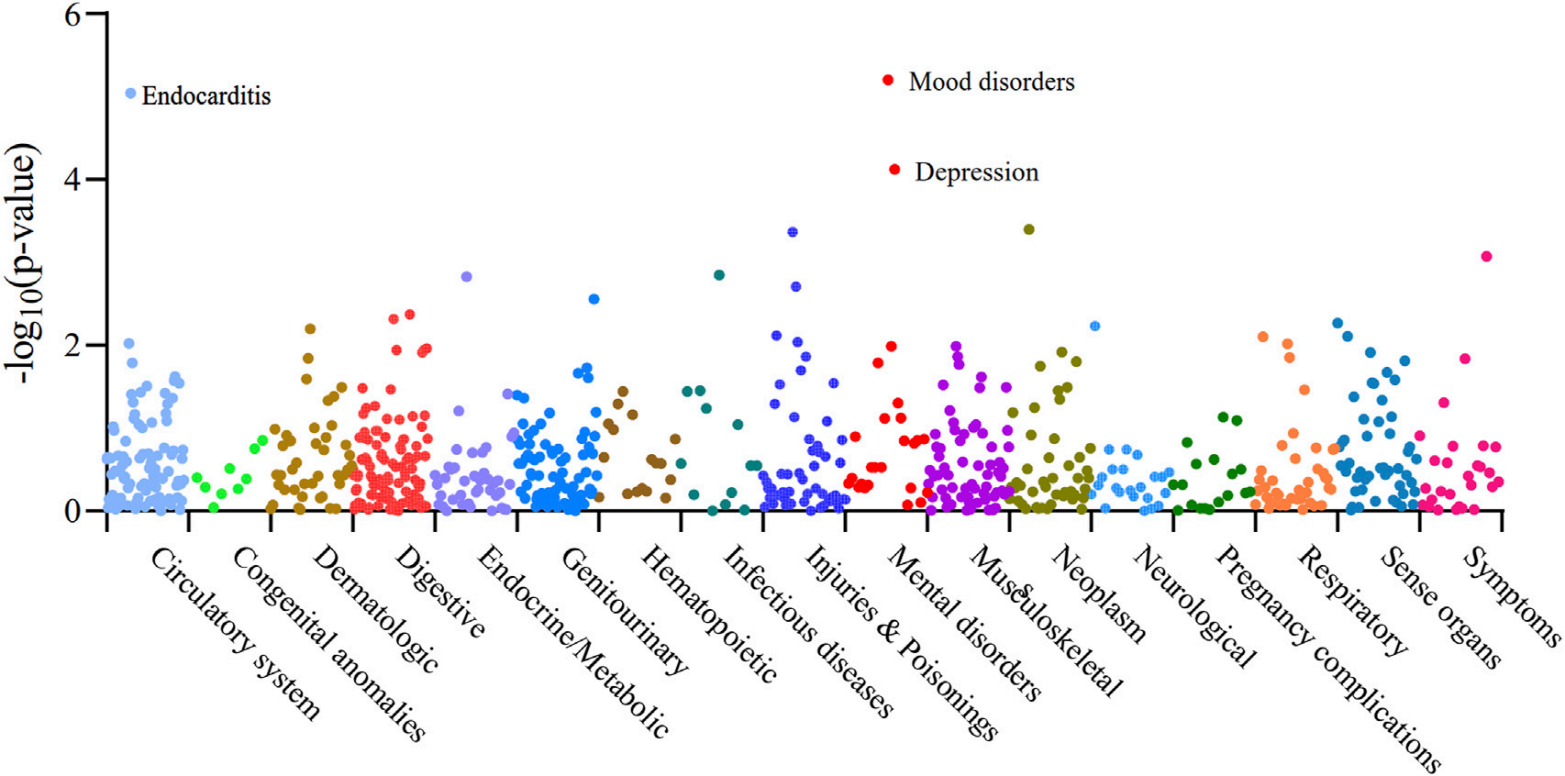
Manhattan plot of the whole phenotype MR results of XBP1. 783 disease phenotypes with case numbers ≥500 were included in the analyses, and these phenotypes were grouped into 17 categories based on disease type. Positive results of the Phenome-wide MR analysis of XBP1 with 783 disease phenotypes were found to be Pfdr < 0.05. Vertical coordinates indicate the *p*-value of the whole phenotype MR results. A dot represents a disease trait, and different colors/dot patterns indicate different disease classifications.

We performed Phenome-wide MR Analysis of XBP1 with 783 non-AF, non-breast cancer phenotypes (Supplementary Table S17). Using the IVW method, we identified a genetic causal effect of XBP1 on three diseases other than AF and breast cancer (Figure 5). With the high expression of XBP1 in blood, it may increase mood disorders (OR = 1.12,95%CI, 1.07–1.18,Pfdr = 0.003),depression (OR = 1.11,95%CI, 1.05–1.17,Pfdr = 0.019) and endocarditis (OR = 1.58,95%CI, 1.29–1.94,Pfdr = 0.003) risk of develop-ment. Thus, the risk of developing each of these genetically linked disorders was reduced when XBP-1 was inhibited with the drug without potential side effects.

## Discussion

After large-scale MR analysis and screening, we found that the correlation between atrial fibrillation and breast cancer is not directly caused by susceptibility between the diseases but rather a causal association established by drugs. We obtained 40 drug targets (23 risk targets and 14 protective targets) for the effects of breast cancer therapeutics on AF. Combined with the C-map database, it was shown that activation of ANXA5 by Docetaxel, inhibition of EIF5A by Fulvestrant, and inhibition of GNA12 by Tamoxifen increased the risk of AF. In contrast, inhibition of ANXA5 by Gemcitabine and Vinorebine and inhibition of PCGF6 by Paclitaxel reduced the risk of AF. As it can act on multiple targets in different tissues, inhibition of MSH6 and SF3B1 by Cyclophosphamide, as well as inhibition of SMAD4 and PSMD2 and activation of ASAH1 and MLST8d by Doxorubicin can have bidirectional effects on AF. In addition, we identified XBP1 as a druggable gene that reduces the risk of AF and breast cancer, and treatment against this target may also reduce other diseases.

The ANXA5 protein, which plays a role in the cell cycle, anti-inflammation, anticoagulation, signaling, and oncology, is encoded by the Annexin A5 (ANXA5) gene (Gerke and Moss, 2002). This gene is currently approved as a target for antitumor drug action (Cannon et al., 2024). Our study suggests a positive correlation between the ANXA5 gene and the occurrence of atrial fibrillation. The ANXA5 protein is one of the membrane-associated proteins with the highest content in cardiac myocytes, primarily located in the T-tubules and sarcomeres of cardiac myocytes. Additionally, this protein is detected in atrial granules and plays a role in regulating the secretion of atrial natriuretic peptide (ANP) (Doubell et al., 1991; Benevolensky et al., 2000). The irregular contraction of the atria due to AF can cause an increase in the level of ANP, while higher levels of ANP cause increased fibrosis in the atria, which in turn causes the maintenance and recurrence of AF (Büttner et al., 2018; van den Berg et al., 2019). Therefore, we hypothesized that the regulation of ANP secretion level is one of the mechanisms by which the ANXA5 gene mediates the development of AF. In addition, the studies have approved that the process of cardiomyocyte apoptosis and myocardial systolic dysfunction involves the role of ANXA5 in regulating of sodium-calcium exchanger 1 (NCX1) activity in cardiomyocytes (Song et al., 1998; Benevolensky et al., 2000; Ravassa et al., 2007; Tritsch et al., 2013). Studies have indicated that dysfunction of NCX1, ryanodine receptor type 2 (RyR2) receptor dysfunction in cardiomyocytes leading to dysregulation of Ca2+ homeostasis will further cause mitochondrial dysfunction, defects in excitation-contraction coupling in cardiomyocytes, which will contribute to the development and maintenance of AF and HF (Lompré et al., 2010; Dridi et al., 2020). Therefore, we hypothesized that the regulation of NCX1 by ANXA5 induces an imbalance of Ca2+ metabolism as an additional mechanism causing AF, and it was hypothesized that ANXA5 could be used as one of the mediators to study the association between AF and HF. Although the current study suggests that the ANXA5 protein has an anti-inflammatory effect, this property is mainly caused by recombinant exogenous membrane-associated protein A5 and is not necessarily shared with cellular membrane-associated protein A5 (Camors et al., 2005). Therefore, more direct studies on the relationship between AF and ANXA5 are necessary.

The mother Against Decapentaplegic Homolog 4 (SMAD4) gene is now widely approved as a therapeutic target for oncology drugs (Zhao et al., 2018; Cannon et al., 2024). The SMAD4 protein is a TGF-β signaling pathway co-mediator SMAD (co-Smad) encoded by the SMAD4 gene and distributed throughout the cytoplasm, and may be involved in the signaling of all TGF-β cytokines. TGF-β is a potent stimulator of collagen production by cardiac fibroblasts, and elevated serum TGF-β levels in patients with AF, as well as increased levels of both TGF-β itself and downstream Smad4 with prolonged duration of AF, allow myocardial fibroblasts to proliferate and promote collagen synthesis, which in turn exerts a pro-fibrotic effect on the atria (Schwarte-Waldhoff and Schmiegel, 2002; Li et al., 2008; Gramley et al., 2010). Atrial structural remodeling caused by myocardial fibrosis is the pathological basis of AF development (Jalife and Kaur, 2015). Our study confirmed that SMAD4 is genetically associated with AF development, which is consistent with the findings of the currently available research evidence. Therefore, we hypothesized that the TGF-β/smad4 signaling pathway, one of the key growth signaling pathways in cardiac fibrosis, is one of the mechanisms that contribute to the development and maintenance of AF by promoting myocardial fibrosis.

Mutations in the splicing factor 3B subunit 1(SF3B1) gene located on the long arm of chromosome 2 (2q33.1) can play a role in the pathogenesis of hematologic tumors, breast cancer, and other tumors by affecting cellular functions and pathways (hemoglobin production, mitochondrial metabolism, and NF-KB pathway, etc.) (Visconte et al., 2012; Zhou et al., 2020; Liu et al., 2021) it has also been approved as a target for antitumor drugs (Cannon et al., 2024). There is still little research evidence on the association of SF3B1 with AF. Still it has been mentioned that clonal hematopoiesis (CHIP) due to SF3B1-containing mutations is closely associated with a pro-inflammatory state and is a novel risk factor for various cardiovascular diseases, including AF (Marnell et al., 2021; Calvillo-Argüelles et al., 2022). Therefore, we hypothesized that SF3B1 mutation-induced immune and inflammatory disorders causing tumor susceptibility may also cause AF development.

Based on the current findings, we have proposed speculations on the target mechanisms that trigger atrial fibrillation (AF). In addition, a few targets were found to be closely related to the occurrence of AF. Among them, the eukaryotic translation initiation factor 5A (eIF5A), a pro-translational protein encoded by the eIF5A gene, is closely related to cellular metabolism, proliferation, differentiation, aging, and mitochondrial function (Barba-Aliaga and Alepuz, 2022; Tan et al., 2010). Studies have confirmed that in an *in vitro* model of malaria infection prepared from human cardiomyocytes, treatment with the inhibitor GC7 reduces the expression level of eIFA5, thereby decreasing the activity of the pro-inflammatory and pro-apoptotic factor, caspase-1, and effectively preventing cardiac injury (Kaiser et al., 2020). The protein encoded by the Polycomb group ring finger 6 (PCGF6) gene, also known as Multivariant binomial logistic regression (MBLR), is an essential regulator of embryonic stem cell pluripotency and induced pluripotent stem cell (iPSC) reprogramming at the transcriptional level (Zdzieblo et al., 2014).

The protein encoded by the PSMD2 gene is one of the important subunits of the 26S proteasome, which plays an important role in protein hydrolysis in the ubiquitin-proteasome system (UPS) (Xiong et al., 2018). Our study suggests that PSMD2 gene expression in cardiac ear tissues and blood is genetically susceptible to AF. The protein encoded by the mammalian lethal with SEC13 protein 8 (MLST8) gene, also known as G protein beta subunit-like (GBL), promotes tumor cell growth and development by regulating mTOR (mechanistic target of rapamycin) signaling and thus does not significantly affect normal cell growth (Kakumoto et al., 2015). Previous studies have only mentioned that MLST8 can mediate hypertrophic cardiomyocyte growth and survival by forming mechanistic target of rapamycin complex 2 (mTORC2) (Balasubramanian et al., 2009). Given that research evidence on the relevance of these genes to cardiovascular disease is still limited and lacks tissue specificity for disease susceptibility, extensive experimental studies are still needed to explore the mechanisms underlying their relevance to AF.

In addition to this, we identified three possible targets for AF protection. The N-acylsphingosine amidohydrolase 1 (ASAH1) gene encodes the acidic ceramidase ASAH1, a glycoprotein with hydrolytic properties, which often acts on the hydrolysis process of ceramide (Cer) to regulate the intracellular homeostasis of Cer and maintain cellular healthy growth (Sugano et al., 2019). Our study provides genetic evidence that ASAH1 gene expression can avoid AF development and that oxidative stress response and inflammatory response have been previously proposed as possible mechanisms for AF development (Rudolph et al., 2010; Hu Y-F. et al., 2015). Therefore, we hypothesized that ASAH1’s role in counteracting cellular oxidative stress and inflammatory regulation is one of the mechanisms to avoid AF occurrence. However, in a study that also explored the mechanisms linking genome-wide association loci to AF risk, ASAH1 was associated with AF susceptibility (Hsu et al., 2018), which is contrary to our findings. It is important to note that the genes for this study were extracted from the left atrial appendage tissue of bi-ethnic subjects from European populations and African Americans. In contrast, our findings, derived from blood tissues and European populations, also did not explore the association of ASAH1 with AF from atrial appendage tissue. Therefore, we hypothesize that tissue specificity of populations and gene expression may contribute to differences in disease susceptibility.

GNA12 encodes a protein belonging to the G12 family of G protein α-subunits, which acts as a signaling molecule in several physiological processes such as anti-inflammatory in cells (Strathmann and Simon, 1991; Yu and Liu, 2023). Given the microenvironmental basis of having an inflammatory environment and macrophage recruitment leading to disease development, we hypothesized that the mechanism by which GNA12 reduces the development of AF has some connectivity to its anti-inflammatory effects (Hu Y-F. et al., 2015; Hulsmans et al., 2023). Notably, GNA12 target was found in the atrial appendage tissue, suggesting tissue specificity in the heart for its action. The MSH6 gene, located on the short arm of chromosome 2, has intrinsic ATPase activity and participates in single base mismatch and insertion-deletion loop repair (Hargreaves et al., 2010). Our study obtained genetic evidence for the protective effect of MSH6 expression against AF in atrial appendage tissue and the blood system. However, the association of MSH6 with cardiovascular disease is still poorly explored, and we cannot speculate whether the possible mechanisms of action are similar to the mechanisms above of tumorigenesis.

X-box binding protein 1 (XBP1) encoded by the XBP1 gene is a unique basic-region leucine zipper (bZIP) transcription factor whose expression level is controlled by endoplasmic reticulum (ER) homeostasis and selective shear response activated (Ono et al., 1991; Wang et al., 2015). The XBP1 gene is currently approved as a drug target for antipsychotic and antimotor disorders in both Fluspirilene and Pimozide (Cannon et al., 2024). We also obtained the result that XBP1 increased the occurrence of psychiatric disorders and depression by Phenome-wide MR Analysis (Cannon et al., 2024). XBP1 promotes tumor cell proliferation and metastasis by modulating immune responses, influencing cell growth and metabolism, and contributing to angiogenesis (Chen et al., 2020). Several studies have confirmed the close association between XBP1 and breast cancer, especially ER-positive breast cancer, which is consistent with our findings. It was noted that XBP1 is highly expressed in dendritic cells (DCs) and is involved in NF-KB signaling, contributing to the IRE1α-XBP1-cMyc axis and the IRE1-XBP1 signaling pathway, and accelerating the expression of Rab9(a small GTPase) and NCOA3, which promotes proliferation, metastasis, and antimmune effects in breast cancer cells (Hu R. et al., 2015; Cubillos-Ruiz et al., 2015; Gupta et al., 2016; Liu Y. et al., 2019; Dong et al., 2019). Therefore, inhibitors against the XBP1 gene target and signaling molecules of the above pathways may be promising tools for developing of novel targeted therapies for breast cancer. Our study demonstrated that XBP1 caused an increased risk of AF, but the evidence to explore the association between XBP1 and AF is currently insufficient. In Phenome-wide MR Analysis, XBP1 was shown to play a role in the risk of endocarditis, and considering that the correlation between AF and infective endocarditis has been pointed out, (Ferrera et al., 2016) we hypothesized that this may be one of the mechanisms by which XBP1 causes the development of AF. In a study exploring the mechanisms of myocardial fibrosis, it was mentioned that Calreticulin overexpression in the endoplasmic reticulum caused disruption of Ca2+ homeostasis, transient activation of the unfolded protein response (UPR)-IRE1a pathway was shown to be a key factor in cardiac fibrosis, and administration of an inhibitor of the UPR was shown to be effective in blocking IRE1α activity and reduced the splicing of XBP1 (Groenendyk et al., 2016). Therefore, the role of spliced XBP1 (the active form of XBP1) in promoting myocardial fibrosis may be one of the mechanisms underlying the development of AF. Our analysis using the C-map database yielded that drug-targeted inhibition of XBP1 had similar effects to knockdown of the gene and that hundreds of drugs with connectivity score >90 were available. In addition, Phenome-wide MR Analysis analysis pointed out that treatments targeting XBP1 did not cause side effects that resulted in the development of other diseases. Genetic evidence of XBP1 in AF and breast cancer provides new ideas for the treatment of both, but a large number of studies are still needed to validate the feasibility and safety of drug-targeted inhibition of XBP1 in the combined state of the two diseases.

Our study is the first to systematically explored the causal relationship between AF and breast cancer in terms of disease and drug action using MR analysis principles and eQTL data, providing genetic support for the association between them. Secondly, we discovered and reported for the first time the effects of the action targets of breast cancer therapeutic drugs on AF and explained the direction of drug action on target genes using the C-map. In addition, we identified the genetic possibilities of breast cancer drugs for the treatment of AF, which contributes to the value of drug utilization. Finally, we explored a common target for the treatment of AF and breast cancer and further analyzed the potential side effects of treatments directed against this target, which has the potential to both reduce the interference of other genes in drug development and provide new targets for therapy in the coexisting state of both diseases. The results of our study are the final results that satisfy the criteria of MR analysis, FDR correction, and colocalization analysis, ensuring reliable and robust results.

However, our study also has some limitations. First, our genetic information data were derived from the European population, limiting the generalization of our findings and requiring further validation for applicability in other populations. Second, we only included clinically used breast cancer drug classes, and not all breast cancer treatment drugs appear in the C-map database, which makes our drug analysis limited. Third, in order to satisfy the independence assumption of MR analysis, we deleted IVs related to LD, and fewer IVs were finally included in the MR analysis, which may affect the credibility of the results. Finally, in conjunction with the currently known research evidence, we were limited in the extent to which we were able to explain the specific mechanisms by which these genes affect AF, and more studies are needed in the future to validate the mechanisms by which these genes affect AF and to determine whether clinical breast cancer drugs can be used for the treatment of AF.

## Conclusion

In this study, we used large-scale MR analysis and thousands of genetic data to reveal the causal relationship between breast cancer therapeutic drugs and AF and finally obtained 40 targets of action of 15 breast cancer therapeutic drugs from blood and/or atrial appendage tissue that affect AF. We evaluated the effects of eight breast cancer therapeutic drugs on AF and preliminarily explained the possible mechanisms of action. Among them, Docetaxel, Fulvestrant, and Tamoxifen increased the risk of AF development, Gemcitabine, Vincristine, and Paclitaxel decreased the risk of AF, and Cyclophosphamide and Doxorubicin could act on multiple targets, thus exerting a bidirectional effect on AF development. In addition, the XBP1 gene was found to be a common druggable target for AF and breast cancer. In conclusion, our study provides genetic evidence for the causal association of AF with breast cancer drugs and new targets for therapy, and future large-scale studies are needed to explore the utility and safety of these targets.

## Data Availability

The datasets presented in this study can be found in online repositories. The names of the repository/repositories and accession number(s) can be found in the article/Supplementary Material.
